# Microwave-Induced Hydrogen Plasma as a New Synthesis Process for High-Entropy Carbides

**DOI:** 10.3390/ma18245520

**Published:** 2025-12-09

**Authors:** Muhammad Shiraz Ahmad, Kallol Chakrabarty, Shane A. Catledge

**Affiliations:** Department of Physics, University of Alabama at Birmingham (UAB), 1300 University Blvd., Birmingham, AL 35294, USA; mahmad2@uab.edu (M.S.A.); kallol89@uab.edu (K.C.)

**Keywords:** high-entropy carbides, microwave-induced hydrogen plasma, plasma-assisted synthesis, nanoindentation, elemental mapping, mechanical properties, equiatomic oxides, ceramic processing

## Abstract

Microwave-Induced Hydrogen Plasma (MIHP) is introduced as a novel synthesis route for producing high-entropy carbides (HECs), offering an alternative to conventional mechanical alloying and/or sintering techniques. In this study, a representative HEC composition, ${\mathrm{MoNbTaVWC}}_{5}$, was successfully synthesized using MIHP processing at 200 Torr. The process employs microwave energy to generate hydrogen plasma to facilitate carbothermal reduction of metal oxide precursors. The plasma environment generates abundant reactive atomic hydrogen species, which enhance reaction spontaneity and promote efficient HEC formation. X-ray diffraction confirmed the formation of a single-phase rocksalt-type face-centered cubic structure. Scanning electron microscopy combined with energy-dispersive X-ray spectroscopy confirmed uniform elemental distribution within the synthesized microstructure. Nanoindentation measurements yielded hardness and elastic modulus values consistent with literature reports for similar compositions. X-ray photoelectron spectroscopy confirmed the chemical state of carbon to be primarily bonded with metals as carbides, with only minor oxygen present as metal-oxides. Raman spectroscopy performed over the 750–1900 ${\mathrm{cm}}^{-1}$ range yielded a featureless spectrum with no detectable D or G bands often observed for sp^2^-hybridized disordered carbon, graphite, or graphene materials. These results validate the structural and chemical purity of the synthesized HECs. This work aims to demonstrate the feasibility and reproducibility of MIHP as a synthesis method for HECs.

## 1. Introduction

High-entropy carbides (HECs) represent an advanced class of ceramics consisting of five or more principal metallic elements in near-equiatomic proportions, forming single-phase solid solutions stabilized by high configurational entropy. These materials exhibit outstanding properties, including high hardness [1,2], excellent thermal stability [3], superior oxidation resistance [4,5], and low thermal conductivity [6], which makes them attractive for applications in extreme environments such as aerospace propulsion, nuclear reactors, and thermal protection systems [7,8,9].

Conventional fabrication techniques, such as hot pressing (HP) and spark plasma sintering (SPS), are predominantly used to consolidate HEC powders into bulk ceramics [10,11,12,13,14,15]. For instance, Feng et al. [12] synthesized dense (Hf–Zr–Ti–Ta–Nb)C ceramics through a two-step process involving carbothermal reduction at 1600 °C, followed by hot pressing at 1900 °C. Similarly, Castle et al. [10] used ball-milled monocarbide powders followed by SPS at temperatures up to 2300 °C and pressures of 16–40 MPa. Despite their effectiveness, these methods are constrained by high energy demands, which stem from peak electrical loads in the tens-of-kilowatts range depending on system configuration and/or milli-step processing routes. Also, resistive heating elements used in conventional furnaces or in sintering equipment can result in substantial thermal energy loss through chamber walls and large contact surfaces with the sample, thereby motivating exploration of more energy efficient processing strategies.

Microwave-induced hydrogen plasma (MIHP) reduction of metal-oxide precursors, as demonstrated in our recent studies, has emerged as a promising cost-effective method for synthesizing advanced materials, including high-entropy borides and alloys [16,17,18,19]. The MIHP approach confines the majority of energy into a small plasma localized in contact with the sample. Minimal energy is conducted or radiated to chamber walls or other non-essential surfaces. Dielectric heating from microwaves occurs throughout the entire volume of the sample, rather than from the outside in, as is the case for conventional heating methods. The dielectric metal oxide precursor powder is particularly efficient in absorbing the microwave energy for heating. Although the sample rests on a ½-inch diameter molybdenum stage, contact is limited to a single face, and the stage remains otherwise electrically and thermally isolated from the reaction site. This configuration minimizes parasitic thermal dissipation.

Beyond spatial confinement and thermal isolation, the efficacy of MIHP also derives from the fundamental interactions between microwave energy and the plasma medium. Microwave energy couples efficiently via electron-impact ionization and dielectric losses, leading to localized heating of the green body while maintaining significantly lower bulk reactor temperatures than conventional furnaces [7]. Energetic electrons in the plasma dissociate molecular hydrogen (H_2_) into atomic hydrogen (H) and other reactive species through electron-impact processes. Atomic hydrogen exhibits significantly greater thermodynamic reduction potential than molecular hydrogen, as evidenced by the following standard Gibbs free energy changes:

$$\begin{array}{cc} \begin{array}{c} MoO_{2}\;+\;4\;H(g)\to Mo\;+\;2\;H_{2}O(g) \end{array},\;\Delta G{}^{\circ}\;\left(298\;K\right) & =\;-736.80\;kJ{\mathrm{mol}}^{-1}\\ \begin{array}{c} {\mathrm{MoO}}_{2}\;+\;2\;H_{2}(g)\to Mo\;+\;2\;H_{2}O(g) \end{array},\;\Delta G{}^{\circ}\;\left(298\;K\right) & =\;76.34\;kJ{\mathrm{mol}}^{-1} \end{array}$$

The positive $\Delta G_{\mathrm{rxn}}^{\circ }$ for molecular hydrogen reduction indicates a non-spontaneous reaction under standard conditions, whereas the highly negative value for atomic hydrogen reflects a thermodynamically favorable pathway. This thermodynamic advantage underscores the efficacy of hydrogen plasma in enabling low-temperature, energy-efficient reduction of refractory metal oxides [20]. Notably, MIHP achieves these critical reactions with peak electrical energy consumption from a MW source as low as 600 Watts.

Despite the compelling advantages of the MIHP approach, it has not been reported for use in the synthesis of HECs. This work addresses the broader need for a scalable, cost-effective, and operationally simplified synthesis route for HECs, which remains unmet by existing furnace-based and SPS processing approaches. ${\mathrm{MoNbTaVWC}}_{5}$ was selected as the reference material due to its high Entropy Forming Ability (EFA) of 125 (eV/atom)^−1^ [7], which predicts intrinsic single-phase FCC stability independent of synthesis route. Accordingly, the objective here is not to demonstrate phase stabilization, but to determine whether MIHP can reliably reproduce the phase purity expected for this composition across independent syntheses.

MIHP process reliability is evaluated through phase and mechanical property reproducibility, supported by X-ray diffraction (XRD) and nanoindentation measurements of hardness and elastic modulus. Elemental uniformity and atomic percentage distribution is assessed through SEM/EDX, while XPS and Raman spectroscopy characterize surface chemistry, carbon bonding, and oxidative behavior. Collectively, these results demonstrate that MIHP offers distinctive processing advantages over conventional synthesis routes and establish it as a cost-effective, energy-efficient, and experimentally reproducible method for producing high-entropy carbides.

## 2. Materials and Methods

The precursor materials for the synthesis of ${\mathrm{MoNbTaVWC}}_{5}$ consisted of high-purity metal oxides—${\mathrm{MoO}}_{3}$, ${\mathrm{Nb}}_{2}O_{5}$, ${\mathrm{Ta}}_{2}O_{5}$, $V_{2}O_{5}$, ${\mathrm{WO}}_{3}$ and graphite (C). All metal oxides were purchased from Nano Research Elements, with stated purities of 99.9% and a mesh size of 325. Graphite (natural, microcrystalline grade, APS 2–15 µm, 99.9995% metals basis) was sourced from Thermo Fisher Scientific Chemicals (Waltham, MA, USA).

The precursors were weighed to obtain an equiatomic Mo-Nb-Ta-V-W ratio in the final carbide, based on the global carbothermal reduction reaction scheme shown below.

(1) $${\mathrm{MoO}}_{3}+\frac{1}{2}{\mathrm{Nb}}_{2}O_{5}+\frac{1}{2}{\mathrm{Ta}}_{2}O_{5}+\frac{1}{2}V_{2}O_{5}+{\mathrm{WO}}_{3}+\frac{37}{2}C\;⟶\;{\mathrm{MoNbTaVW}(C)}_{5}+\frac{27}{2}\mathrm{CO}.$$

This equation serves solely as a stoichiometric reference to ensure equiatomic metal ratios in the precursor mixture and is not intended to represent the mechanistic complexity of the MIHP process. The plasma environment contains a spectrum of reactive hydrogen and oxygen-based species—ranging from atomic and molecular hydrogen to charged ions, free electrons, and oxidizing radicals—that collectively enable complex reduction dynamics and intermediate-phase formation.

Each metal oxide exhibits distinct vapor pressure and volatilization behavior, influenced by local temperature and chamber pressure during processing. As temperature increases, transient intermediates—such as suboxides or intermetallics—may form before consolidating into the final high-entropy carbide phase. Similar transitions were observed in our plasma-assisted synthesis of high-entropy borides [16]. Consequently, Equation (1) cannot predict the full reaction pathway under these highly non-equilibrium conditions. Empirical tracking of structural evolution is therefore necessary to elucidate the actual synthesis mechanism.

The mixed precursor powders were homogenized by ball milling in a SPEX SamplePrep 8000M Mixer/Mill, (Metuchen, NJ, USA) using a WC-lined vial with dimensions of 2.25 in. diameter × 2.5 in. length. Dry milling was performed for 2 h using two WC balls (7/16 in. diameter), followed by wet milling in acetone for 4 h using two ${\mathrm{ZrO}}_{2}$ balls (1/2 in. diameter). Wet milling in acetone facilitates improved powder dispersion and helps suppress particle agglomeration, thereby promoting more uniform mixing and compositional homogeneity [21]. A 10 min cooling interval was introduced after each hour of milling to prevent excessive temperature rise. The mill operated along a figure-8 trajectory to promote uniform mixing and particle refinement.

**Figure 1:**
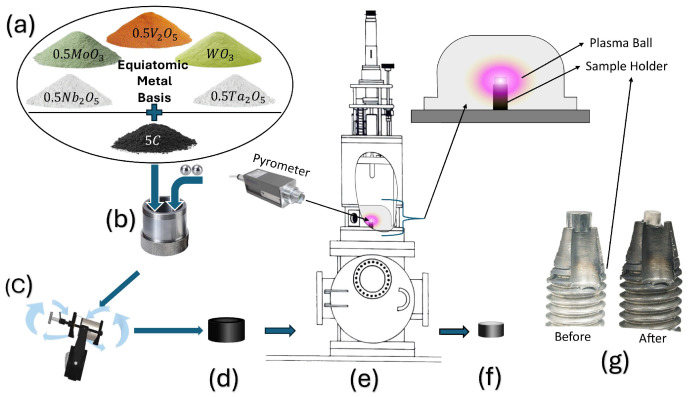
Schematic of the experimental procedure: (**a**) precursor mixing, (**b**,**c**) ball milling, (**d**) green pellet formation, (**e**) MIHP processing, (**f**) final synthesized sample, (**g**) before and after MIHP processed sample on sample holder.

Green compacts were prepared by uniaxial pressing approximately 300 mg of milled powder into disk-shaped pellets (3.5 mm height, 5 mm diameter) under an applied load of approximately 30 MPa. The final HEC pellet mass was approximately 150 mg, reflecting 50% mass loss due to oxide-to-carbide reduction. The complete experimental sequence is illustrated schematically in Figure 1.

The equipment used for MIHP processing included a bell-jar-type MPCVD chamber (Wavemat™ MPDR 313EHP, Wavemat Inc., Plymouth, MI, USA) featuring a 5-inch quartz bell jar diameter, powered by a 2.45 GHz, 0.6–5 kW microwave power supply (Sairem™, GMP60KSM, Neyron, France). Key process parameters included chamber pressure (controlled by a throttling valve), gas flow rate, and microwave power. The monitored parameter included the sample temperature (measured by an optical pyrometer). The minimum instrumental microwave power of 0.6 kW was found to be sufficient to ignite the plasma, and minimum chamber pressure to reach optimum temperature for HEC was experimentally determined to be 200 Torr. The green pellet was placed on a molybdenum stage; the chamber was evacuated to 0.167 Torr, and backfilled with 500 sccm argon to establish a stabilized plasma at 5 Torr. The gas then transitioned into pure hydrogen and the pressure was ramped to 200 Torr over 10 min. When the sample temperature, exceeded 1900 °C, a 90 min dwell time was initiated, during which the temperature stabilized at 2100 ± 100 °C and the pressure at 200 Torr.

X-ray diffraction (XRD) was used to determine the crystal structure via a Malvern Panalytical Empyrean diffractometer (Malvern Panalytical B.V., Almelo, The Netherlands), equipped with Cu Kα radiation (λ=1.54186 Å), operated at 45 kV and 40 mA. Data were collected using a step size of 0.0131° and a counting time of 16.32 s per step. The polishing of the MIHP-processed samples was performed using a MetPrep grinding and polishing system. The initial grinding was carried out with 320-grit SiC paper until surface flatness was achieved, followed by 600-grit SiC paper for 2 min. Subsequent polishing steps employed a 6 µm diamond suspension for 2 min, 1 µm diamond suspension for 5 min, and a final polish with 0.02 µm colloidal silica for 15 min.

Microstructural examination and elemental mapping were carried out using a field emission scanning electron microscope (FEI Quanta FEG 650, Hillsboro, OR, USA) equipped with an energy-dispersive X-ray spectroscope (EDX), operated at an accelerating voltage of 20 kV.

Nanoindentation testing was performed using an Agilent Nano Indenter G200 system (MTS Nano Instruments, Oak Ridge, TN, USA) equipped with a Berkovich diamond indenter and operated in continuous stiffness measurement (CSM) mode. Instrument calibration was conducted using a fused silica reference standard with an accepted Young’s modulus of 72 ± 1 GPa, both before and after testing the sample. For each sample, an average of seven indents were performed to ensure statistical reliability of the measured mechanical properties. Re-evaluation of the fused silica reference post-indentation confirmed that the tip geometry remained unchanged throughout the experiments and that calibration accuracy was maintained.

XPS analysis was performed using Phi Electronics Versaprobe 5000 (Chanhassen, MN, USA) equipped with a micro-focused monochromatic Al Kα source (λ=1486.6 eV). The sample surface was cleaned by ${\mathrm{Ar}}^{+}$ ion milling for 14 min. There was no strong enough peak for adventitious carbon after milling, and binding energy calibration was carried out using the reported C 1s peak positions for ${\mathrm{Ar}}^{+}$-milled binary carbides MoC, NbC, TaC, VC, and WC [22]. The average C 1s value of 283.11 eV obtained from these references was used as the internal calibration point for carbon bonded to metals in the HEC, and all spectra were shifted accordingly. Raman spectra were collected using a Dilor XY micro-Raman spectrometer (Lille, France) with a 532 nm laser, a 1200 groove/mm grating, and a 100× objective.

## 3. Results and Discussion

### 3.1. Phase Formation and Reproducibility

**Figure 2:**
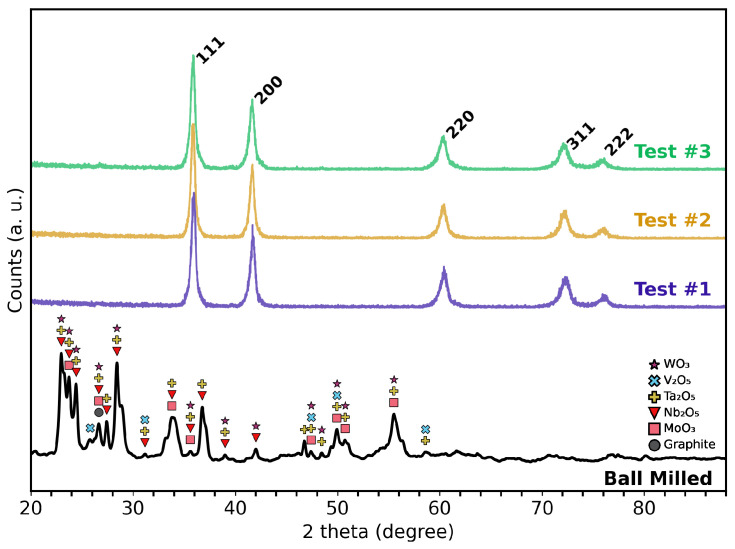
XRD patterns showing the powder after ball milling (performed at ambient temperature). “Test #1” to “Test #3” are the three independently synthesized ${\mathrm{MoNbTaVWC}}_{5}$ HEC samples processed via MIHP at approximately 2100 °C. The HEC patterns confirm single FCC phase formation and reproducibility across all test samples.

The single-phase FCC structure, expected for the ${\mathrm{MoNbTaVWC}}_{5}$, was confirmed for each of the three independently synthesized test samples, as shown in Figure 2. Rietveld refinement of the XRD data verified that the material adopts a rock salt-type structure with space group $Fm\bar{3}m$. No evidence of residual crystalline oxides or graphite were detected by XRD. The lattice parameters computed for Test #1 through Test #3 were 4.343(0)Å, 4.337(0)Å, and 4.340(0)Å, respectively. These values fall within the reported range of 4.34–4.35Å for ${\mathrm{MoNbTaVWC}}_{5}$ [23,24,25].

### 3.2. Elemental Uniformity

**Figure 3:**
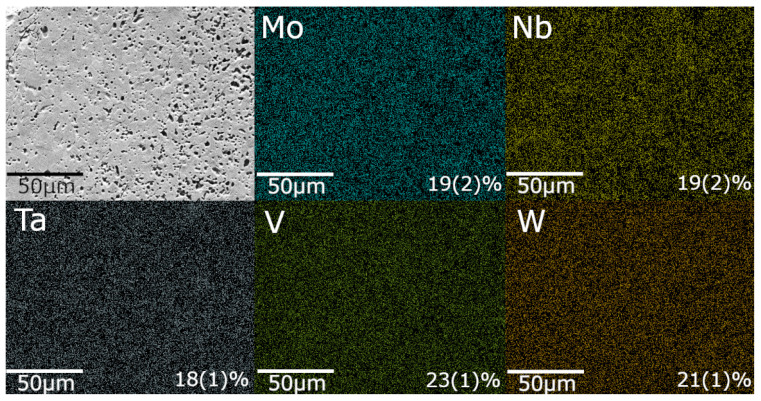
SEM image (top left) and elemental distribution maps for Mo, Nb, Ta, V, and W in a ${\mathrm{MoNbTaVWC}}_{5}$ sample synthesized at 200 Torr. Atomic percentages are indicated in the respective elemental maps.

Scanning electron microscopy (SEM) was performed using the Quanta 650 FEG (FEI, Hillsboro, OR, USA), coupled with energy-dispersive X-ray spectroscopy (EDX), and EDX analysis was used to evaluate the surface morphology and elemental distribution in the synthesized ${\mathrm{MoNbTaVWC}}_{5}$ samples. As shown in Figure 3, the EDX elemental maps confirm a near-equiatomic distribution of Mo, Nb, Ta, V, and W, consistent with the nominal equimolar design (∼20 at.% each). The measured compositions deviate by no more than ±3 at.% from the nominal value, which is within the expected compositional uncertainty derived from propagated measurement errors during EDX analysis.

The MIHP-processed samples exhibited a relative density of 56.0%, corresponding to 44% total porosity, including 11.8% open pores, determined according to ASTM C373-18. Surface morphology and pore distribution are visually evident in the SEM micrograph shown in Figure 3. This value is substantially lower than the 98–99% relative density (corresponding to 1–2% porosity) typically reported for the same HEC composition when consolidated by conventional SPS processing [26,27], where simultaneous uniaxial pressure and a high applied current facilitate particle rearrangement, enhance mass transport, and collapse residual pore networks. Because MIHP operates without external pressure and at lower chamber pressures, gases produced during rapid oxide-to-carbide conversion are less effectively expelled, leading to higher porosity.

### 3.3. Nanoindentation Hardness and Modulus

**Figure 4:**
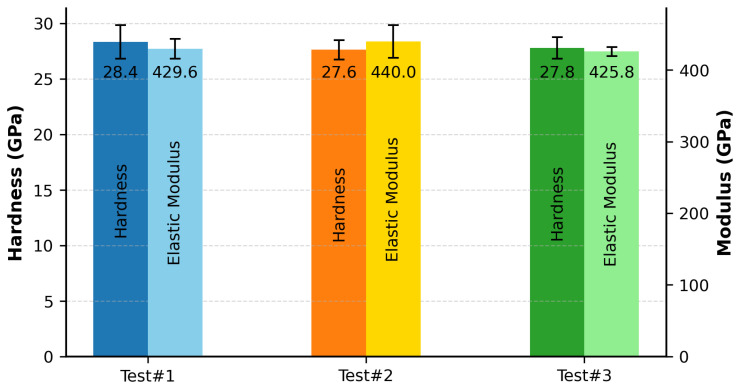
Nanoindentation hardness and elastic modulus for ${\mathrm{MoNbTaVWC}}_{5}$ samples synthesized via MIHP.

Nanoindentation measurements were performed using a Berkovich diamond indenter calibrated using a fused silica standard. Figure 4 presents the averaged hardness and elastic modulus values, determined at an indentation depth of 100 nm.

The nanohardness values measured in this study—28.35 ± 1.51 GPa, 27.63 ± 0.87 GPa, and 27.80 ± 0.96 GPa for Test #1, Test #2, and Test #3, respectively—fall well within experimentally reported values for this HEC class, ranging from 25.1 to 31.69 GPa [23,25,26,27,28]. While all reported values are categorized as nanohardness, variations in measurement parameters—such as indentation load, tip geometry, and penetration depth—are either unspecified or inconsistent across studies, thereby limiting the scope for strict quantitative comparison.

The corresponding elastic modulus values were determined to be 429.6 ± 14.0 GPa, 440.0 ± 22.8 GPa, and 425.8 ± 6.1 GPa for Test #1, Test #2, and Test #3, respectively. These were compared with the theoretical modulus of 459 GPa predicted by the AFLOW (ROM) computational framework, as reported by Sarker et al. [24]. The calculated deviations for the three tests—6.39%, 4.14%, and 7.22%—all fall within the generally accepted ±10% range, indicating close agreement between experimental results and theoretical predictions.

Furthermore, the elastic modulus values measured in this study fall within the broader spectrum of experimentally reported values for this HEC class, ranging from 366.35 to 551 GPa [23,25,27,28]. Chen et al. [28] observed that microstructural parameters—particularly grain size and pore population—have an impact on mechanical properties, leading to slight reductions in nanohardness and more pronounced, often abrupt, variations in elastic modulus. This correlation explains the relatively narrow dispersion in hardness values and the substantially wider spread in modulus data reported across the literature and corroborated in the present work.

Scanning Electron Microscopy (SEM) imaging (Figure 3) revealed the presence of porosity in the MIHP-processed samples. Although the present study did not investigate the individual contributions of microstructural or thermal factors, the measured mechanical properties are consistent with the empirical trends reported in the HEC literature. Specifically, nanohardness values across this material class tend to exhibit relatively narrow dispersion despite compositional and processing differences, whereas elastic modulus values show broader variability and often differ from theoretical predictions due to intrinsic microstructural or methodological factors.

### 3.4. Carbon Bonding Analysis

**Figure 5:**
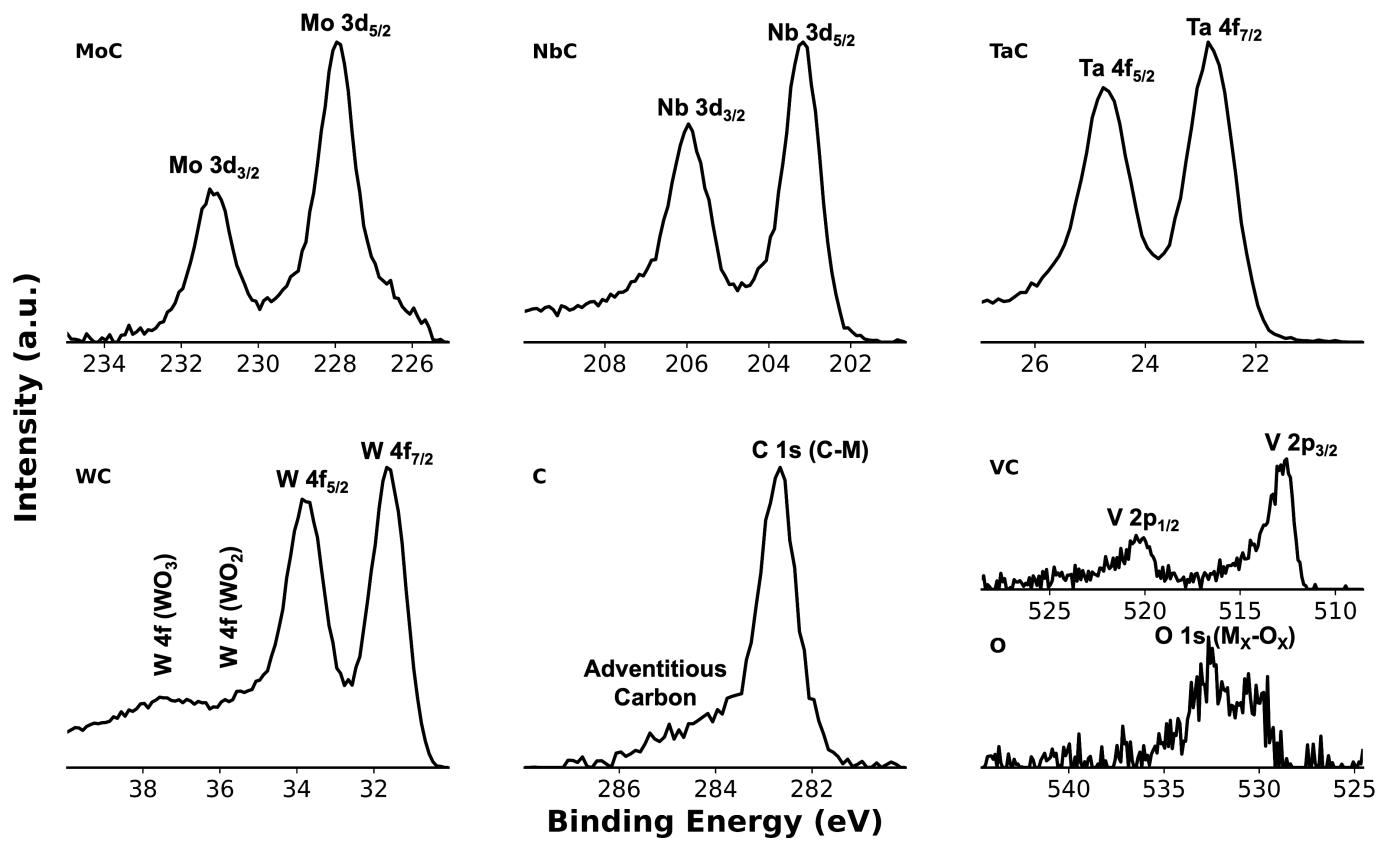
High-resolution XPS spectra of the ${\mathrm{MoNbTaVWC}}_{5}$ sample highlighting the C 1s binding energy region corresponding to the carbon bonded to metals. Metal–carbon peaks represent the core levels of constituent metals chemically bonded to carbon within the HEC structure.

In the high-resolution XPS spectra (Figure 5), the dominant C 1s (C–M) peak was fixed at 283.11 eV using carbide reference binding energies. The weak shoulder at a higher binding energy, centered near 284.31 eV, is attributed to surface adventitious carbon. The O 1s feature is attributed to weak oxidation from ambient exposure [29].

Since no XPS data have been previously reported for the exact ${\mathrm{MoNbTaVWC}}_{5}$ composition, direct spectral comparison is not possible. Nonetheless, core-level binding energies reported in Table 1 align with reported values for chemically analogous HEC systems, including (HfNbTaTiZr)C, (HfNbTiVZr)C, (MoNbTaTiV)C, (WTaNbZrTi)C, and (TiVNbMoW)C [29,30,31,32,33]. For example, Ta ${4f}_{7/2}$ (23.22 eV), Nb ${3d}_{5/2}$ (203.61 eV), Mo ${3d}_{5/2}$ (228.36 eV), W ${4f}_{7/2}$ (32.02 eV), and V ${2p}_{3/2}$ (513.08 eV) all fall within the commonly reported ±0.5 eV range. This consistency across distinct HEC chemistries supports the calibration procedure and confirms chemically bonded metal–carbide states in the synthesized ${\mathrm{MoNbTaVWC}}_{5}$. Quantitative peak-area analysis yielded C 1s (45%), W 4f (10%), Ta 4f (9%), Mo 3d (9%), Nb 3d (9%), V 2p (5%), and O 1s (9%).

Because XPS is a surface-sensitive and semi-quantitative technique with a sampling depth of only ∼5–10 nm, the survey scan primarily reflects near-surface chemistry rather than the bulk composition. The material had been stored in ambient laboratory conditions prior to analysis, so the observed O 1s signal may potentially originate from surface oxidation. This interpretation aligns with the XRD results in Figure 2, which probe the full sample volume and show only a single-phase rocksalt HEC structure with no detectable secondary crystalline phases, suggesting that any oxygen-bearing species are restricted to the surface or are negligible in the bulk. Accordingly, the high-resolution C 1s and metal core-level binding energies support metal–carbide bonding consistent with the expected HEC chemistry.

Raman spectroscopy was conducted over the 750–1900 ${\mathrm{cm}}^{-1}$ range to detect the presence of any residual unreacted graphite from the precursor powder or to identify ${\mathrm{sp}}^{2}$-hybridized intermediate structures. Specifically, neither the D-band (∼1350 ${\mathrm{cm}}^{-1}$) nor the G-band (∼1580 ${\mathrm{cm}}^{-1}$) was detected, signifying the absence of ${\mathrm{sp}}^{2}$-hybridized disordered carbon. The Raman spectra were featureless, as expected for chemically disordered HECs [30], and are therefore not shown here.

## 4. Limitations and Future Work

In the current MIHP configuration, one face of the green body rests directly on the molybdenum sample holder, which prevents direct plasma interaction with the bottom surface. As a result, the lower region undergoes slower reduction and carbide formation than the plasma-exposed surface, requiring longer annealing times to achieve full conversion. Addressing this contact-induced shielding effect will be an important design priority for future reactor iterations.

The synthesized HECs exhibit markedly higher porosity and lower relative density than SPS-processed counterparts. Although porous ceramics have documented functional applications [34,35], controlling porosity was not an objective of the present work. Preliminary observations suggest that increased chamber pressure enhances densification, indicating a tunable parameter that warrants systematic investigation.

Additionally, the 90-min synthesis duration was selected conservatively to ensure complete phase formation rather than to define an optimized processing condition. Establishing the minimum required processing time will require quantitative assessment of plasma heating efficiency, absorption depth, and reaction kinetics. Planned studies will map these relationships to determine accelerated yet complete synthesis windows.

## 5. Conclusions

Collectively, the structural, chemical, and mechanical results confirm that MIHP reliably synthesizes single-phase ${\mathrm{MoNbTaVWC}}_{5}$. XRD verifies a rocksalt FCC structure across independently processed samples, while SEM/EDX mapping shows near-equiatomic elemental distribution consistent with the configurational entropy-driven phase stabilization expected for HECs. High-resolution XPS and Raman analyses support metal–carbide bonding with only minor surface oxidation from ambient exposure and no detectable ${\mathrm{sp}}^{2}$ carbon, consistent with bulk XRD showing no dominant secondary phases. The nanoindentation hardness and modulus fall within the established range for this composition, indicating successful phase formation despite the higher porosity associated with low-pressure MIHP processing. Taken together, these observations establish MIHP as a reproducible and experimentally consistent synthesis route capable of producing chemically and structurally uniform high-entropy carbides.

Beyond establishing synthesis reliability, MIHP provides distinct processing advantages over SPS and furnace-based routes. Reactive atomic hydrogen increases the effective chemical potential for oxide reduction, enabling rapid carbide formation, while microwave-selective, volumetric heating concentrates energy directly within the green body rather than the chamber. These coupled effects reduce temperature requirements and electrical power demand, offering a potentially more energy-accessible pathway. The comparatively higher porosity is plausibly linked to gases becoming trapped within the compact during rapid solid-solution formation under low-pressure MIHP conditions, limiting their ability to diffuse out before densification. Thus, MIHP represents a scalable and energy-efficient processing alternative for future HEC development.

## Figures and Tables

**Table 1:** Core-level binding energies measured for MoNbTaVWC_5_.

Peak Label	Binding Energy (eV)
C 1s (C–M, carbide, fixed)	283.11
Mo ${3d}_{5/2}$ (Mo–C)	228.36
Nb ${3d}_{5/2}$ (Nb–C)	203.61
Ta ${4f}_{7/2}$ (Ta–C)	23.22
W ${4f}_{7/2}$ (W–C)	32.02
W ${4f}_{7/2}$ (${\mathrm{WO}}_{2}$)	34.83
W ${4f}_{7/2}$ (${\mathrm{WO}}_{3}$)	38.03
V ${2p}_{3/2}$ (V–C)	513.08
V ${2p}_{1/2}$ (V–C)	520.77
V ${2p}_{3/2}$ (substoichiometric/onset oxide)	514.24

## Data Availability

The original contributions presented in this study are included in the article. Further inquiries can be directed to the corresponding author.
